# High-dose insulin and dexamethasone combined with radiotherapy in endometrial stromal sarcoma recurring with multiple metastases: A case report

**DOI:** 10.1097/MD.0000000000033525

**Published:** 2023-04-14

**Authors:** Libin Liang, Jun Wang, Jing Xie, Yanping Xu, Lansen Zhang, Dehui Liu, Xinglong Tong

**Affiliations:** a Xinglong Institute of Pharmaceutical and Medical Science, Shijiazhuang, China; b Department of Radiotherapy, the Fourth Hospital of Hebei Medical University, Shijiazhuang, China; c Graduate school of Hebei Medical University, Shijiazhuang, China.

**Keywords:** case report, dexamethasone, endometrial stromal sarcoma, insulin, radiotherapy

## Abstract

Endometrial stromal sarcoma (ESS) is a rare malignant tumor. There is insufficient data supporting the efficiency of current treatments in multiple metastatic settings, and novel therapeutic options for ESS are considered an area of high unmet clinical need.

**Patient concerns:**

We report the case of a 28-year-old woman who was diagnosed with ESS after undergoing total hysterectomy and left adnexectomy at another hospital. Two years later, the disease recurred, with multiple abdominal cavities and lung metastases. The patient was treated with a variety of chemotherapeutic drugs, including tyrosine kinase inhibitors, at the same hospital; however, none of them inhibited disease progression.

**Diagnoses:**

Computed tomography (CT) revealed multiple masses in the abdominal and pelvic cavities and multiple pulmonary nodules. Ultrasound-guided biopsy was performed and the tumor tissue was histologically confirmed after treatment.

**Interventions:**

Insulin 300–400 IU was administrated by intravenous infusion in 10% glucose (500 mL) with disodium adenosine triphosphate 60 mg, coenzyme A 100 units, 10% potassium chloride 5 mL and 25% magnesium sulfate 5 mL. Dexamethasone (20–25 mg/d) was diluted with 10 mL of 2% lidocaine and then intraperitoneally injected after ascites draw. After 9 months, the patient was referred to another center for radiotherapy.

**Outcomes:**

CT images tomography showed recurrent pelvic masses, and multiple abdominal cavity and lung metastases gradually shrunk with treatment. Histological biopsy revealed growth arrest of tumor cells. The patient experienced for 3-years survival.

**Lessons:**

High-dose insulin and dexamethasone combined with radiotherapy provides a novel and promising option for patients with multiple ESS metastases.

## 1. Introduction

Endometrial stromal sarcoma (ESS) is a rare malignant tumor that constitutes approximately 0.2% of all uterine malignancies and 10% of uterine sarcomas.^[1]^ Surgery is the primary therapy for ESS.^[2,3]^ However, approximately one-third to one-half of the patients with ESS develop recurrence or metastasis. Currently, there is no standard therapy for patients with recurrent disease. Beyond surgery, various treatment modalities like sex hormone therapy, chemotherapy and targeted drugs, radiotherapy, or a combination of these have been used to treat recurrent ESS.^[3,4]^ Due to the large variation in pathologic characteristics, combined with the scarcity of patients, there is insufficient data supporting the efficiency of current treatments in multiple metastatic settings, and novel therapeutic options for ESS are considered an area of high unmet clinical need.^[3]^

Herein, we report a patient with endometrial stromal sarcoma recurring with multiple abdominal cavities and lung metastases. After chemotherapy failures at other hospitals, we treated the patient with high-dose insulin and dexamethasone combined with radiotherapy on the basis of supportive care, and the patient survived for over 3-years survival. We hope that this case report will play a role in establishing an effective treatment strategy for ESS.

## 2. Case presentation

**Figure 1.**
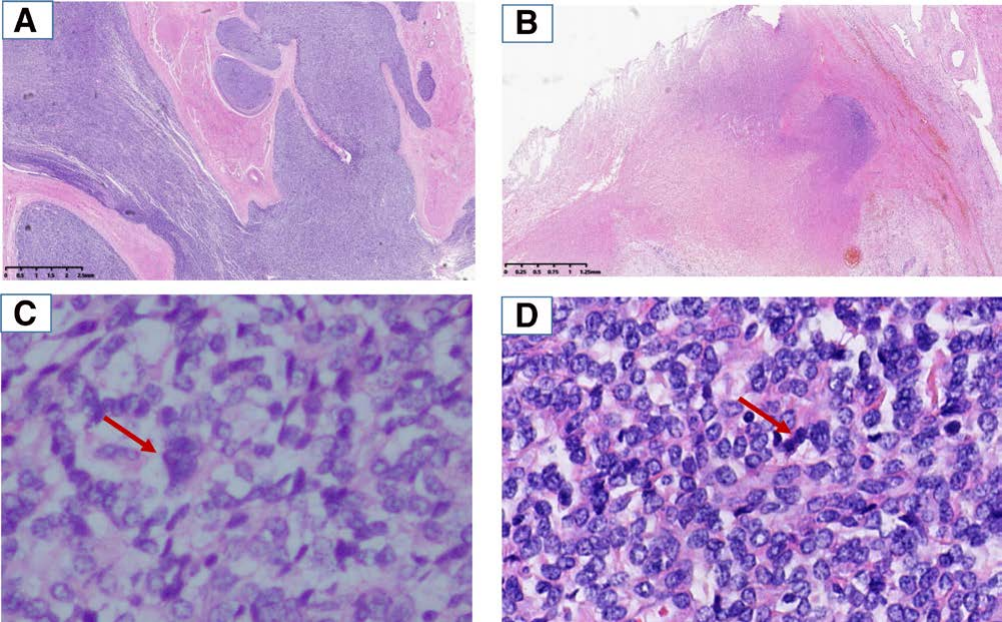
Hematoxylin and eosin staining of the resected primary uterine tumor. (A) Tumor tissue showing extensive invasion and destruction of myometrium (×100). (B) There was massive necrosis in tumor tissue (×100). (C) Tumor cells showing few cytoplasm, vacuolated nucleus with visible nucleolus. The arrow points to a giant tumor cell (×400). (D) Mitotic cells were visible as shown by the arrow (×400).

A female patient, born in 1988, P2L2, underwent total hysterectomy and left adnexectomy in August 2016 (at the age of 28) at a local hospital. Histopathologically, the tumor presented as a solid nest, with extensive invasion and destruction of the myometrium (tongue-like invasion of the myometrium) accompanied by hemorrhage and necrosis. The intravascular emboli grew along the vessels of the left cornu uteri. Microscopically, the tumor cells were round, large in size, with few cytoplasms, vacuolated nuclei with visible nucleoli, and occasionally giant (Fig. 1). Immunohistochemistry indicated that the tissue sample was positive for vimentin, CD10, CD 34, and Ki-67 (30–40%) and negative for CK and desmin. The pathological diagnosis was ESS.

Two years after the initial surgery (2018), the patient was diagnosed at the local hospital as postoperative recurrence with multiple abdominal cavity and pulmonary metastasis since magnetic resonance imaging (MRI) showed multiple nodules in both lungs, multiple nodules in the retroperitoneum at the level of the upper pole of the kidney, multiple parenchymal lesions in the pelvic cavity, posterior bladder, left pelvic inlet. The patient was treated with various chemotherapeutic drugs, including pezopanib, paclitaxel, apatinib mesylate, triaprilizumab, and carelizumab, during the second half of 2018 and the first half of 2019; however, the condition was not effectively controlled. The patient presented to our clinic on September 16, 2019 because of progressive illness.

**Figure 2.**
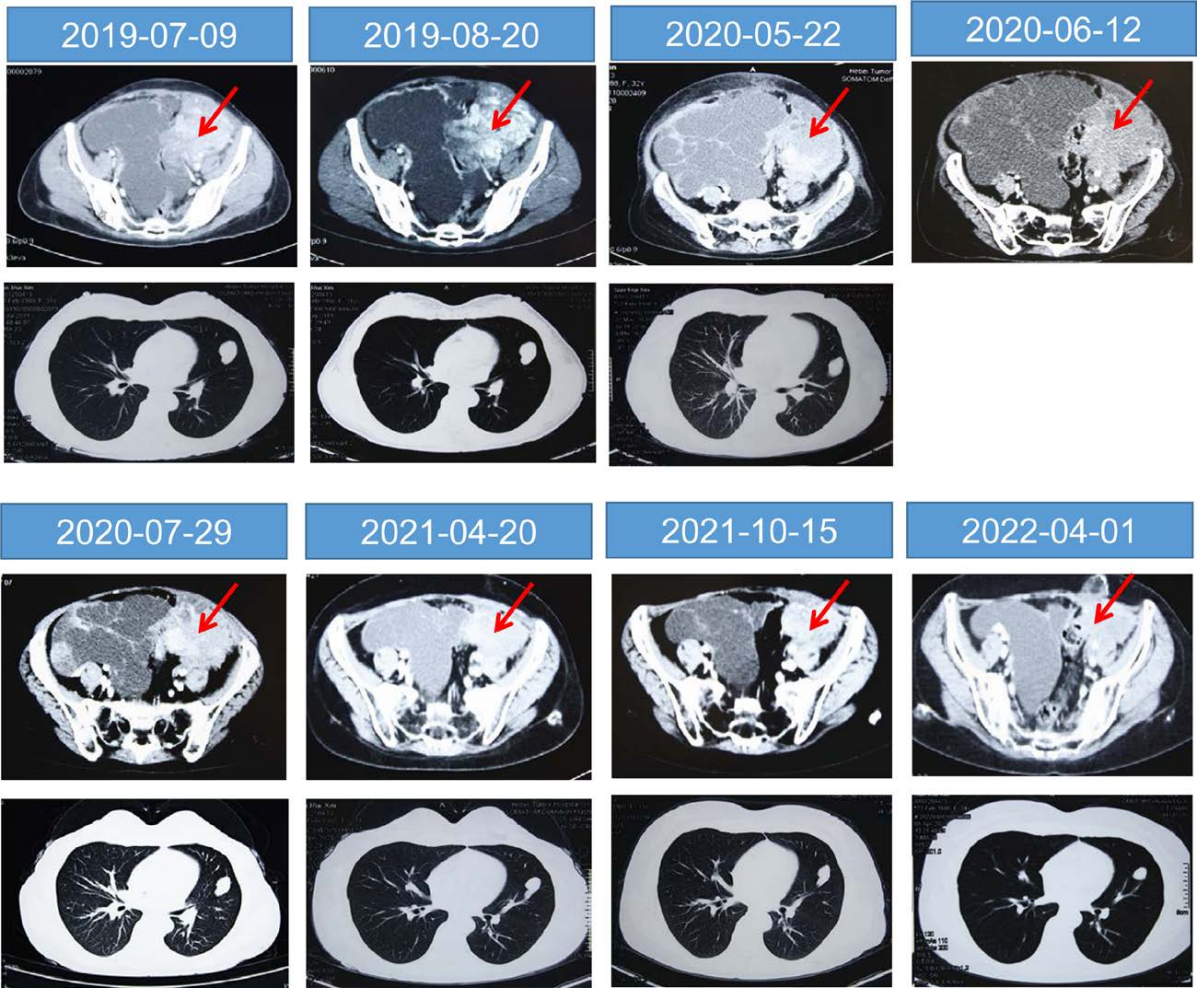
Computed tomography scan showing the changes of recurrent space-occupying lesion (as shown by the arrows) in abdominal cavity (upper panels) and the metastatic tumors in lung (lower panels) during the treatment.

General examination revealed that the patient looked pale and extremely weak, and even a little activity that caused wheezing and palpitations. The blood pressure was 80/40 mm Hg, heart rate 106/min and respiratory rate 50/min. Eyelids and both lower limbs showed edema, particularly in the ankle. The abdomen was obviously bulging, with varicose veins, and an abdominal circumference of 98 cm. Abdominal examination revealed a palpable mass measuring approximately 9 × 7 cm on the left margin of the pelvis. Computed tomography (CT) (2019-8-20) (Fig. 2) showed multiple masses in the abdominal and pelvic cavities, among which a mass at the left margin of the pelvis was obviously enlarged compared with the CT images taken on 2019-07-09. Multilocular cystic lesions with large amounts of fluid were detected in the abdominal cavity. Multiple enlarged lymph nodes were also observed in the abdominal cavity and retroperitoneum. A filling defect in the inferior vena cava was observed, likely due to tumor thrombus formation. Additionally, multiple nodules were observed in both the lungs. The patient was diagnosed with postoperative recurrence of ESS with multiple abdominal cavities and lung metastases. Massive ascites caused by peritoneal metastasis and vena cava tumor thrombus led to hypovolemia.

Our therapeutic plan included various supportive treatments, such as human albumin infusion to correct the hypovolemic status, interferon to increase immunity activity, and maintenance of electrolyte balance. The main medication regimen included insulin (300–400 IU per day or every other day) and dexamethasone (20–25 mg/d). Insulin was administered via intravenous infusion in 500 mL of 10% glucose with disodium adenosine triphosphate 60 mg, coenzyme A 100 units, 10% potassium chloride 5 mL, and 25% magnesium sulfate 5 mL. Acsitic fluid was drawn from multiple sites every day, with 10–30 mL of ascitic fluid at each site. Dexamethasone was diluted with 10 mL of 2% lidocaine, 10 mg of anisodamine hydrochloride was added, and the solution was intraperitoneally injected after ascites was drawn. After 1 month of continuous treatment, the patient’s general condition improved, and blood pressure, heart rate, and other vital indicators returned to normal. The ascites gradually reduced with abdominal circumference from 98 to 86 cm. The patient was able to take care of herself in daily life. After prolonged treatment, insulin was administered every 3 to 7 days. Ascitites were drawn, and dexamethasone was intraperitoneally injected every other day or every few days. Various supportive nutritional treatments and electrolyte imbalance corrections were continued. The abdominal circumference dropped to78 cm and the patient was able to perform general housework activities. At 9 (2020-05-22) and nearly 10 months (2020-06-12) after treatment, CT images showed that metastases in the left pelvic margin shrank slightly, while there was no significant change in enlarged lymph nodes in the abdominal cavity and retroperitoneum and metastases in the lungs. These findings suggest that the combination of high-dose insulin and dexamethasone effectively blocks tumor growth.

Considering that the patient’s condition was stable and general condition was good, the patient was referred to another center for radiotherapy (June 10, 2020–July 16, 2020). The abdominal irradiation regimen was 95% PTV ≥ 32.4 GY 18.5, once every other day and 3 times a week with a total of 12 doses of radiation. Radiotherapy induced thrombocytopenia and anemia in the patient at a later stage, and thus platelet-raising drugs, together with plasma and blood components, were supplied. After 1 month of radiotherapy, the ascites further reduced with the abdominal circumference down to 70. After 3 months, the patient could drive by herself and return to work as a kindergarten teacher. Chest and abdominal CT examinations (July 29, 2020, April 20, 2021, October 15, 2021, and April 1, 2022) showed that the metastatic tumors in multiple sites within the lungs were gradually reduced, and the multilocular cystic lesions in the abdominal cavity became smaller. In the later stage. Except for intermittent administration of calcium and calcitriol to treat osteoporosis and colchicine to fight high uric acid, no other drugs were used. The patient has lived in good condition for over 3 years.

**Figure 3.**
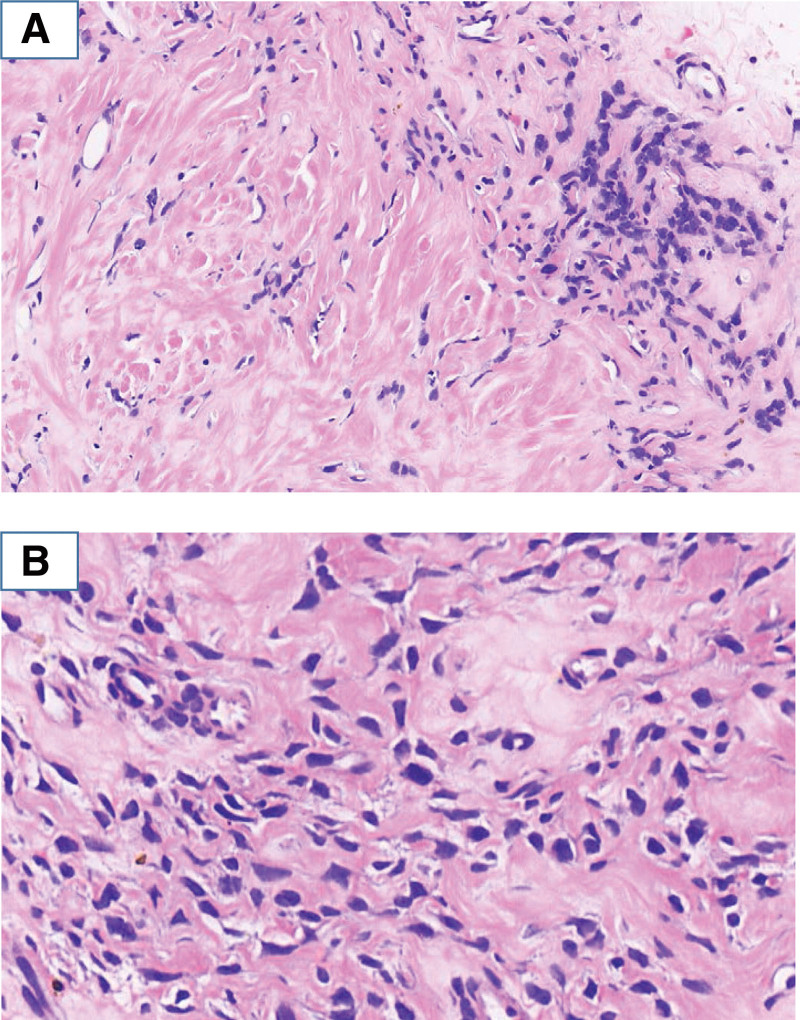
Hematoxylin and eosin staining of the puncture tumor tissue after combined treatment. (A) Tumor tissue showing fibrosis and sclerosis scattered in the tumor cells (×200). (B) Tumor cells showing nuclei pyknosis without nucleolus (×400).

On June 2, 2022, an ultrasound-guided biopsy of the tumor tissue in the right abdominal cavity was performed for histopathology. Histopathological examination revealed that the tissue was fibrotic and hyaline, with occasional residual tumor cells at the edge, and the tumor nuclei were pyknotic and hyperchromatic (Fig. 3). It suggests that the growth of tumor is in a state of being suppressed.

## 3. Discussion

The current classification of ESS includes low-grade and high-grade ESS.^[5]^ The pathological retrorespective analysis and clinical features, including early postoperative recurrence and multiple metastases, accompanied by the fact that metastases are not sensitive to various chemotherapeutic drugs, are in favor of high-grade ESS.

At present, the treatment of ESS with multiple metastases includes chemotherapy and sex hormone and radiotherapy.^[6,7]^ None of the chemotherapy drugs, including tyrosine-kinase inhibitors, significantly inhibited disease progression before the patient came to our clinic. Data have shown that the combination of radiotherapy and chemotherapy may improve prognosis.^[8]^ However, the initial physical condition of the patient was unable to tolerate radiotherapy. Therefore, in addition to the essential supportive treatment, the patient was mainly treated with high-dose insulin combined with dexamethasone. The results showed that the growth of the tumor was arrested, accompanied by a reduction in ascites and gradual improvement in the patients’ physical constitution. Radiotherapy was performed to further reduce the tumor size. Although the metastasis did not completely disappear, the patient survived and returned to normal life.

The idea of insulin plus dexamethasone for cancer treatment came about after an accidental “mistake” in which a patient with advanced cancer misused a high-dose of insulin and his condition improved obviously. The potential adverse reactions of high-dose insulin are hypoglycemia and hypokalemia, which are rare in practice because of concomitant glucose and potassium supplementation. Both disorders were easily reversed by glucose and electrolyte replacements. In recent years, high-dose insulin (1–10 IU/kg/h) with adapted glucose supplementation has been used to treat calcium-channel blocker poisoning, and high doses of up to 22 IU/kg/h have been shown to be safe.^[9]^ The likely antitumor mechanism of high-dose insulin is unknown. Experimental studies have found that insulin is a broad-spectrum inducer of stem cell differentiation. Because tumor cells and stem cells have highly similar biological characteristics, we hypothesized that insulin may inhibit proliferation and induce differentiation of tumor cells. However, the specific mechanism needs to be studied further. Dexamethasone regulates a variety of tumor-associated signaling pathways by binding to glucocorticoid receptors, which may affect the proliferation, growth, and metastasis of tumor cells through anti-inflammatory and anti-angiogenesis effects.^[10,11]^

Currently, patients with high-grade ESS experience earlier and more frequent recurrences and are at a higher risk of death due to the disease.^[8]^ High-dose insulin and dexamethasone combined with radiotherapy provides a novel and promising option for patients with multiple ESS metastases. The mechanism underlying the therapeutic effectiveness is worthy of further study.

## Author contributions

**Conceptualization:** Xinglong Tong.

**Data curation:** Libin Liang.

**Project administration:** Libin Liang, Jun Wang, Yanping Xu, Lansen Zhang, Dehui Liu.

**Software:** Jing Xie.

**Supervision:** Xinglong Tong.

**Writing – original draft:** Libin Liang.

**Writing – review & editing:** Xinglong Tong.
